# Aminoglycoside Resistance Rates, Phenotypes, and Mechanisms of Gram-Negative Bacteria from Infected Patients in Upper Egypt

**DOI:** 10.1371/journal.pone.0017224

**Published:** 2011-02-17

**Authors:** Gamal F. Gad, Heba A. Mohamed, Hossam M. Ashour

**Affiliations:** 1 Department of Microbiology and Immunology, Faculty of Pharmacy, Minia University, Minia, Egypt; 2 Department of Microbiology and Immunology, Faculty of Pharmacy, Cairo University, Cairo, Egypt; 3 Eugene Applebaum College of Pharmacy and Health Sciences, Wayne State University, Detroit, Michigan, United States of America; National Institute of Allergy and Infectious Diseases, National Institutes of Health, United States of America

## Abstract

With the re-emergence of older antibiotics as valuable choices for treatment of serious infections, we studied the aminoglycoside resistance of Gram-negative bacteria isolated from patients with ear, urinary tract, skin, and gastrointestinal tract infections at Minia university hospital in Egypt. *Escherichia coli* (mainly from urinary tract and gastrointestinal tract infections) was the most prevalent isolate (28.57%), followed by *Pseudomonas aeruginosa* (25.7%) (mainly from ear discharge and skin infections). Isolates exhibited maximal resistance against streptomycin (83.4%), and minimal resistance against amikacin (17.7%) and intermediate degrees of resistance against neomycin, kanamycin, gentamicin, and tobramycin. Resistance to older aminoglycosides was higher than newer aminoglycoides. The most common aminoglycoside resistance phenotype was that of streptomycin resistance, present as a single phenotype or in combination, followed by kanamycin-neomycin as determined by interpretative reading. The resistant *Pseudomonas aeruginosa* strains were capable of producing aminoglycoside-modifying enzymes and using efflux as mechanisms of resistance. Using checkerboard titration method, the most frequently-observed outcome in combinations of aminoglycosides with β-lactams or quinolones was synergism. The most effective combination was amikacin with ciprofloxacin (100% Synergism), whereas the least effective combination was gentamicin with amoxicillin (53.3% Synergistic, 26.7% additive, and 20% indifferent FIC indices). Whereas the studied combinations were additive and indifferent against few of the tested strains, antagonism was never observed. The high resistance rates to aminoglycosides exhibited by Gram-negative bacteria in this study could be attributed to the selective pressure of aminoglycoside usage which could be controlled by successful implementation of infection control measures.

## Introduction

Aminoglycosides are broad-spectrum antibiotics of high potency that have been traditionally used for the treatment of serious Gram-negative infections [1]. They kill bacteria by inhibiting protein synthesis via binding to the 16S rRNA and by disrupting the bacterial cell membrane integrity [2]. The year 1944 marked the beginning of the aminoglycoside era with streptomycin being introduced, and was followed by the discovery of a series of milestone aminoglycosides such as kanamycin, gentamicin, and tobramycin [3]. The semi-synthetic aminoglycosides, dibekacin, amikacin, and netilmicin, which were introduced in the seventies, allowed clinicians to overcome the anti-aminoglycoside resistance acquired by some bacterial strains against some earlier aminoglycosides [3]. There has been a recent trend towards using older antibiotics, such as aminoglycosides in the management and treatment of serious infections that are difficult to treat [4]. The reason is that the relative low-level use of older antibiotics could have contributed to preservation of activity against many bacterial isolates, which are becoming more resistant to newer more-frequently used antibacterial agents [4]. Thus, in this study we explored the aminoglycoside resistance patterns of bacterial isolates of patients with different infectious diseases at Minia university hospital in Egypt.

There are several mechanisms that contribute to the development of aminoglycoside resistance. These include the deactivation of aminoglycosides by aminoglycoside-modifying enzymes which act on specific sites of the aminoglycosides causing acetylation via aminoglycoside acetyltransferase (AAC), adenylation via aminoglycoside nucleotdyltransferase (ANT) or phosphorylation via aminoglycoside phosphotransferase (APH) [2], [5], [6]. This is a major mechanism by which clinical isolates of Gram-negative and Gram-positive bacteria cause an enzymatic modification of the amino or hydroxyl groups aminoglycosides, causing them to bind poorly to ribosomes and thus fail to trigger energy-dependent phase II, which allows bacteria to survive [7]. Other mechanisms include the reduction of the intracellular concentration of aminoglycosides by changes in the outer membrane permeability which is usually a non-specific resistance mechanism, inner membrane transport, active efflux or drug trapping, the alteration of the 30S ribosomal subunit target by mutation, and finally methylation of the aminoglycoside binding site [2]. The efflux system has been specifically shown to be involved in aminoglycoside resistance in *Pseudomonas aeruginosa* infections in several countries [8], [9], [10]. Thus, we tested if this resistance mechanism was operational in the isolates.

Antimicrobial synergy resulting from combination antibiotic therapy has been a preferred therapeutic approach to treat serious bacterial infections by broadening antibacterial spectrum and preventing the development of resistance. Thus, we tested if aminoglycosides still retained their characteristic ability of producing synergistic bactericidal activity against *Escherichia coli* isolates in combination with antibiotics inhibiting cell wall biosynthesis, such as β-lactams [11].

## Materials and Methods

### Sample collection

A total of 250 clinical samples were examined; 115 from patients with ear infections (purulent ear discharge), 66 from urine of patients with urinary tract infections, 44 from patients with skin infections (wounds, abscesses, and burn exudates), and 25 from stool of patients with Gastrointestinal tract infections were collected from patients at Minia University Hospital in Egypt in the period from 2007 to 2009. Among these, 175 Gram-negative bacterial isolates were identified and recovered using standard microbiological procedures. Moreover, samples were examined for *P. aeruginosa* by polymerase chain reaction, as described previously [12], [13], [14].

Minia University Hospital is a major hospital in Upper Egypt and is the only academic hospital in Minia governorate (population of about 4 millions). It serves patients referred to it from other smaller hospitals in Minia and its surrounding towns and villages in Upper Egypt.

### Ethics Statement

Ethical approval to perform the study was obtained from the institutional review board of Minia University. Written informed consent was obtained from all patients included in the study.

### Antimicrobial susceptibility

Both agar dilution and disk diffusion methods were used to test the susceptibility of the strains to streptomycin (Sigma-Aldrich, USA), neomycin (Sigma), kanamycin (Sigma), gentamicin (Sigma), and amikacin (Bristol-Myers Squibb, USA) according to Clinical and Laboratory Standards Institute procedures, 2007 [15]. Disks for streptomycin (10 µg), neomycin (30 µg), kanamycin (30 µg), gentamicin (10 µg), amikacin (30 µg), and tobramycin (10 µg) (Oxoid, UK) were used in the disk diffusion method.

### Interpretative reading

By testing the susceptibility of the isolates against a range of aminoglycoside antibiotics, determining the aminoglycoside resistance patterns can be used to infer resistance phenotypes and possible aminoglycoside modifying enzymes. This method has been referred as interpretative reading [16].

### Detection of aminoglycoside-modifying enzymes by agar diffusion

We used a protocol modified from Benveniste and Davies's method [17]. Briefly, a crude enzyme extract was prepared from the aminoglycoside-resistant *Pseudomonas aeruginosa* strain (S40). For a total of 50 µl reaction mixture, 25 µl of the enzyme extract (containing 1 mg/ml protein) was incubated in the presence of 22 nmoles of the tested aminoglycoside antibiotics, 50 nmoles dithiotheriotol (Sigma), 2.5 µmoles tris pH 8.1 (Sigma), 120 nmoles ATP (Sigma) and 0.4 µmoles Magnesium chloride (BDH, UK). To monitor the extent of inactivation of the antibiotics, 10 µl of the reaction mixture (with and without the enzyme extract) was spotted on filter paper disks, which were placed on Mueller-Hinton agar plates seeded with a sensitive standard *Pseudomonas aeruginosa* PAOI strain. Plates were incubated at 37°C for 24 hr. In case of inactivation of the antibiotics, the presence of a crude enzyme extract reduces the inhibition zone of antibiotics than the inhibition zone in negative control disks in the absence of the enzyme extract (normally more than 5 mm).

### Detection of the efflux system

The minimum inhibitory concentrations (MIC) of 25 MDR *Pseudomonas aeruginosa* isolates were examined against streptomycin, gentamicin, neomycin, kanamycin, and amikacin in the presence or absence of 50 µM of the efflux inhibitor carbonyl cyanide-m-chlorophenylhydrazone (CCCP) (Sigma) [18]. The reduction in MIC of an antibiotic with CCCP is indicative of the presence of an efflux system mode of resistance against this antibiotic.

### Evaluation of antibiotic combination

In order to determine the potential presence of synergy that can help overcome bacterial resistance, we used the checkerboard titration method [19]. The aim was to test combinations of aminoglycosides (Gentamicin and Amikacin) with β-lactams and quinolones by broth microdiluion on 15 *Escherichia coli* strains. The MIC of each of gentamicin and amikacin was determined before and after combination with β-lactams (Amoxicillin, Cephradine, and Cefotaxime) or quinolones (Ciprofloxacin). The fractional inhibitory concentrations (FIC) were calculated as the MIC of drug A and drug B in combination divided by the MIC of drug A or drug B. The FIC index specifies whether the combination had a synergistic, additive, indifferent, or antagonistic effect. Synergism was defined as an FIC index of less than 0.5. Additive or indifferent effects were defined as an FIC index of 0.5–4 (Additive 0.5–1; Indifferent 1–4). Antagonism was defined as an FIC index of more than 4 [20]. The following antibiotics were tested at the following concentrations: Gentamicin (2 to 1024 µg/ml); Amikacin and Cephradine (0.5 to 256 µg/ml); Ciprofloxacin, Amoxicillin, and Cefotaxime (0.5 to 128 µg/ml).

### Statistical Analysis

Statistical analysis was performed using the SPSS software (Version 16). Chi-square test was used to compare between agar dilution and disk diffusion methods in classifying the Gram-negative isolates in the resistant or the susceptible category. P values of less than 0.05 were considered to be statistically significant.

## Results

As shown in Table 1, the biochemical identification of a total of 175 Gram-negative bacterial isolates indicated the presence of 50 isolates of *Escherichia coli* (50/175, 28.57%), 45 isolates of *Pseudomonas aeruginosa* (25.7%), 17 isolates of *Serratia marcescens* (9.7%), 16 isolates of *Proteus mirabilis* (9.14%), 13 isolates of *Enterobacter aerogene*s (7.42%), 8 isolates of *Proteus vulgaris* (4.57%), 8 isolates of *Klebsiella pneumonia* (4.57%), 8 isolates of *Morganella morganii* (4.57%), 6 isolates of *Citrobacter freundii* (3.43%), and 4 isolates of *Citrobacter koseri* (2.29%). The 50 isolates of *Escherichia coli* included 25 from urine, 6 from ear discharge, 6 from skin infections, and 13 from stool. The 45 isolates of *Pseudomonas aeruginosa* included 10 from urine, 25 from ear discharge, 9 from skin infections, and one from stool. *Escherichia coli* was the most prevalent Gram-negative bacteria in urinary tract and gastrointestinal tract infections, whereas *Pseudomonas aeruginosa* was the most prevalent in ear discharge and skin infections.

**Table 1:** Prevalence of the isolated microorganisms in different sample sites.

Total number of isolates (250)	Gastrointestinal Tract Infections (25)	Skin Infections (44)	Ear Infections (115)	Urinary Tract Infections (66)	
50	13 (52%)	6 (13.64%)	6 (5.22%)	25 (37.88%)	*Escherichia coli*
45	1 (4%)	9 (20.45%)	25 (21.7%)	10 (15.15%)	*Pseudomonas aeruginosa*
17	0 (0%)	6 (13.64%)	4 (3.47%)	7 (25.75%)	*Serratia marscecence*
16	0 (0%)	1 (2.27%)	15 (13.04%)	0 (0%)	*Proteus mirabilis*
13	0 (0%)	2 (4.55%)	2 (1.74%)	9 (13.63%)	*Enterobacter aerogenes*
8	0 (0%)	0 (0%)	8 (6.95%)	0 (0%)	*Proteus vulgaris*
8	0 (0%)	3 (6.82%)	2 (1.74%)	3 (4.55%)	*Klebsiella pneumonia*
8	0 (0%)	1 (2.27%)	2 (1.74%)	5 (7.58%)	*Morganella morgani*
6	0 (0%)	1 (2.27%)	4 (3.47%)	1 (1.52%)	*Citrobacter freundii*
4	0 (0%)	2 (4.55%)	2 (1.74%)	0 (0%)	*Citrobacter koseri*
175	14	31	70	60	Total

The isolated Gram-negative bacteria showed variable degrees of resistance to aminoglycosides as assessed by agar dilution and disk diffusion methods (Tables 2 and 3). Isolates exhibited maximal resistance against streptomycin (83.4%), and minimal resistance against amikacin (17.7%) and intermediate degrees of resistance against neomycin, kanamycin, gentamicin, and tobramycin. Chi-square test revealed that there was no significant difference between agar dilution and disk diffusion methods in classifying the Gram-negative isolates in the resistant or the susceptible category (P>0.05). It is noteworthy that, with the exception of *Serratia marcescens*, resistance of Gram-negative bacteria to tobramycin was less than resistance to gentamicin.

**Table 2:** Resistance patterns of Gram-negative bacteria to different aminoglycosides (Agar Dilution method).

Amikacin	Gentamicin	Kanamycin	Neomycin	Streptomycin	No. of isolates	Isolates
2 (4%)	20 (40%)	23 (46%)	27 (54%)	39 (78%)	50	*Escherichia coli*
13 (28.8%)	32 (71.1%)	41 (91.1%)	40 (88.9%)	42 (93.3%)	45	*Pseudomonas aeruginosa*
3 (17.6%)	7 (41.1%)	11 (64.7%)	10 (58.8%)	13 (76.4%)	17	*Serratia marcescens*
1 (6.3%)	2 (12.5%)	8 (50%)	6 (37.5%)	12 (75%)	16	*Proteus mirabilis*
6 (46.2%)	8 (61.5%)	9 (69.2%)	9 (69.2%)	11 (84.6%)	13	*Enterobacter aerogene*s
2 (25%)	5 (62.5%)	7 (87.5%)	6 (75%)	7 (87.5%)	8	*Proteus vulgaris*
2 (25%)	4 (50%)	7 (87.5%)	6 (75%)	6 (75%)	8	*Klebsiella pneumonia*
1 (12.5%)	5 (62.5%)	5 (62.5%)	4 (50%)	8 (100%)	8	*Morganella morganii*
1 (16.6%)	3 (50%)	3 (50%)	4 (66.7%)	5 (83.3%)	6	*Citrobacter freundii*
0 (0%)	2 (50%)	3 (75%)	3 (75%)	3 (75%)	4	*Citrobacter koseri*
31 (17.7%)	88 (50.3%)	117 (66.9%)	115 (65.7%)	146 (83.4%)	175	Total

*Breakpoints were: ≥16 µg/ml for streptomycin and neomycin, ≥25 µg/ml for kanamycin, ≥8 µg/ml for gentamicin, and ≥32 µg/ml for amikacin according to CLSI (2007).

Interpretative reading was used to detect phenotype patterns of aminoglycoside resistance in Gram-negative bacteria as well as possible modifying enzymes (Table 4). The isolates were classified as wild-type classical strains (strains which are susceptible to all aminoglycosides with no acquired resistance mechanisms) and resistant strains (data not shown). The resistant strains were phenotyped according to their susceptibility patterns. As expected, different isolates exhibited different phenotypes. However, there were general trends that were consistent in all isolates. For example, the most common resistance phenotype in the isolates was that of streptomycin, which was present as a single phenotype or in combination, followed by kanamycin-neomycin.

In order to study resistance mechanisms in more detail, we decided to focus on *Pseudomonas aeruginosa* isolates, given the scarcity of data on the antimicrobial resistance mechanisms of *Pseudomonas aeruginosa* in Egypt. Results indicated that the diameters of the inhibition zones of both gentamicin and amikacin against the sensitive *Pseudomonas* strain PAO1 were reduced by the addition of a crude enzyme extract prepared from a resistant *Pseudomonas aeruginosa* strain (S40) as compared to the diameters of inhibition zones around control disks that were not treated with the enzyme extract (data not shown). This showed that production of aminoglycoside-modifying enzymes was one of the mechanisms of *Pseudomonas aeruginosa* resistance to aminoglycosides in this study. Moreover, when using the efflux inhibitor CCCP, the minimum inhibitory concentrations (MICs) of aminoglycosides (streptomycin, gentamicin, neomycin, kanamycin, and amikacin) against *Pseudomonas aeruginosa* isolates were reduced indicating that efflux is a second leading mechanism of *Pseudomonas aeruginosa* resistance to aminoglycosides in this study (Table 5).

One of the common ways to overcome bacterial antibiotic resistance is the use of antibiotic combinations. In order to evaluate the effects of combination of gentamicin and amikacin with cefotaxime, cephradine, amoxicillin, and ciprofloxacin, we employed the checkerboard titration method (Table 6). Synergism was the most frequently observed outcome in all antibiotic combinations against the tested strains. The most effective combination was amikacin with ciprofloxacin (Synergism in all isolates), whereas the least effective combination was gentamicin with amoxicillin (Synergistic FIC indices in only 8 isolates; 53.3%, additive FIC indices in 4 isolates; 26.7%, and indifferent FIC indices in 3 isolates; 20%). Antagonism was not observed with any combination. The studied combinations were additive and indifferent against few of the tested strains (Table 6).

## Discussion

Given the challenge of having very few newly-discovered antibiotics in recent years and the scarcity of any recent novel developments in the design of molecules that can inhibit the resistant bacterial enzymes, we intended to re-evaluate the effectiveness of aminoglycosides in the treatment of different bacterial infections. Aminoglycosides are older antibiotic compounds that, due to a general low-level use in recent years, are claimed by some to have preserved their activities against some of the most resistant hard-to-treat bacterial infections [4]. Moreover, there have been reports of the effectiveness of altering the administration dosages of aminoglycosides and/or the use of aminoglycoside analogues in reducing their typical adverse effects, such as nephro- and ototoxicity, while still preserving reasonable antibacterial activities [21], [22], [23], [24]. To the best of our knowledge, this is the first study on aminoglycoside resistance in Upper Egypt.

As evident in Tables 2 and 3, resistance to older aminoglycosides such as streptomycin, neomycin, and kanamycin was generally higher than resistance to newer aminoglycoides with broader spectra of antibacterial activity [11], such as gentamicin, amikacin, and tobramycin. This suggests that the implementation of newer aminoglycosides could still be the gold standard for treatment in cases of more resistant bacteria and could be especially useful in cases of mixed infections.

Amikacin is a derivative of kanamycin A with the amino group at position 1 acylated by 4-amino-2-hydroxybutyrate [25]. In this study, amikacin was more active against Gram-negative bacteria than other tested aminoglyside antibiotics (Tables 2 and 3), which is consistent with results from other studies [24], [26]. The high activity of amikacin observed in this study may be attributed to the presence of the aminohydroxybutyryl group, which generally prevents the enzymatic modification of amikacin at multiple positions without interfering with binding to the A site of rRNA [25].

**Table 3:** Resistance patterns of Gram-negative bacteria to different aminoglycosides (Disk Diffusion method).

Tobramycin	Amikacin	Gentamicin	Kanamycin	Neomycin	Streptomycin	No. of isolates	Isolates
15 (30%)	8 (16%)	18 (36%)	22 (44%)	24 (48%)	41 (82%)	50	*Escherichia coli*
10 (22.2%)	9 (20%)	25 (55.6%)	36 (80%)	35 (77.8%)	40 (88.9%)	45	*Pseudomonas aeruginosa*
4 (23.5%)	1 (5.9%)	3 (17.6%)	9 (52.9%)	10 (58.8%)	12 (70.7%)	17	*Serratia marcescens*
2 (12.5%)	1 (6.25%)	2 (12.5%)	8 (50%)	8 (50%)	13 (81.3%)	16	*Proteus mirabilis*
5 (38.5%)	4 (30.8%)	6 (46.2%)	8 (61.5%)	8 (61.5%)	10 (76.9%)	13	*Enterobacter aerogene*s
3 (37.5%)	2 (25%)	4 (50%)	7 (87.5%)	7 (87.5%)	8 (100%)	8	*Proteus vulgaris*
4 (50%)	2 (25%)	5 (62.5%)	6 (75%)	5 (62.5%)	7 (87.5%)	8	*Klebsiella pneumonia*
2 (25%)	1 (12.5%)	3 (37.5%)	5 (62.5%)	5 (62.5%)	6 (75%)	8	*Morganella morganii*
1 (16.7%)	1 (16.7%)	2 (33.3%)	3 (50%)	3 (50%)	4 (66.7%)	6	*Citrobacter freundii*
0 (0%)	0 (0%)	0 (0%)	2 (50%)	3 (75%)	2 (50%)	4	*Citrobacter koseri*
46 (26.3%)	30 (17.1%)	68 (38.8%)	106 (60.6%)	108 (61.7%)	143 (81.7%)	175	Total

It is noteworthy that other researchers have detected much higher rates of resistance of Gram-negative bacteria to amikacin in Turkey (49.7%) and in India (55.1%) [27], [28]. However, these resistance rates were still lower than resistance rates to tobramycin in Turkey (82.4%) and India (83.6%) [27], [28]. Resistance to gentamicin varied in different countries with very high resistance rates (94.5%) reported in Turkey compared to much lower resistance rates (32.6%) reported in India [27], [28].

Although the AAC(6′)-I enzyme was absent or rarely found in most isolates in this study (Table 4), it was the most prevalent enzyme in other studies [28]. The synthesis of more than one enzyme simultaneously led to various composite phenotypes that increased the number of resistant strains that we observed. In this study and in other studies, the most frequent resistance phenotype was that of streptomycin for *Escherichia coli*, *Klebsiella pneumonia*, and *Proteus species* [29]. On the other hand, the most common resistance phenotype for *Enterobacter species* in a study performed in Greece was that of kanamycin-neomycin [29]. The different resistance patterns in different geographical regions may be related to differences in antibiotic (aminoglycoside) treatment regimens.

**Table 4:** Aminoglycoside resistance phenotype and mechanism of Gram-negative bacteria (Interpretative Reading method).

Pseudomonas aeruginosa	Morganella morganii	Klebsiella pneumonia	Citrobacter species	Enterobacter aerogenes	Proteus species	Serratia marcescens	Escherichia coli	Mechanism	Aminoglycoside Resistance Phenotype
73.33%	62.5%	62.5%	50%	46.2%	75%	70.6%	62%	ANT(3″)- I	S
62.22%	50%	37.5%	40%	30.85%	50%	52.9%	20%	APH(3′)- I	K, Nm
2.22%	12.5%	25%	0%	7.7%	8.33%	17.6%	8%	ANT(2″)- I	K, T, G
48.9%	12.5%	12.5%	10%	7.7%	8.33%	0%	2%	AAC(3′)- I	G
4.44%	0%	0%	0%	0%	0%	5.9%	0%	AAC(6′)- I	T, A
15.6%	12.5%	25%	10%	30.8%	12.5%	0%	20%	Impermeability	S, K, Nm, G, T, A

*S, Streptomycin; K, Kanamycin; Nm, Neomycin; G, Gentamicin; T, Tobramycin; A, Amikacin.

AAC, Acetyltransferase; ANT, nucleotidyltransferase or adenylyltransferase; APH, Phosphotransferase

The high resistance rates to aminoglycosides exhibited by Gram-negative bacteria in this study could be attributed to the selective pressure of aminoglycoside usage [2]. Resistance of *Pseudomonas aeruginosa* to antipseudomonal aminoglycosides, such as gentamicin, tobramycin, and amikacin has been reported in most parts of the world [30]. Data indicating negative effects of CCCP on resistance of selected *Pseudomonas aeruginosa* isolates (Table 5) is consistent with data from recent studies on *Pseudomonas aeruginosa* resistance patterns in Egypt [12].

**Table 5:** Effect of adding CCCP on antibiotic resistance pattern of *Pseudomonas aeruginosa* isolates.

Amikacin		Gentamicin		Kanamycin		Neomycin		Streptomycin		Isolate number
MIC in presence of 10 µM CCCP (mg/L)	MIC (mg/L)	MIC in presence of 10 µM CCCP (mg/L)	MIC (mg/L)	MIC in presence of 10 µM CCCP (mg/L)	MIC (mg/L)	MIC in presence of 10 µM CCCP (mg/L)	MIC (mg/L)	MIC in presence of 10 µM CCCP (mg/L)	MIC (mg/L)	
0.5	4	2	16	8	32	16	32	4	32	1
0.25	0.5	2	128	8	64	64	128	8	64	2
2	16	2	512	8	512	32	512	8	1024	3
0.5	2	1	32	4	32	4	64	4	64	4
4	32	0.5	16	4	32	4	32	8	1024	5
4	16	2	32	8	128	8	128	8	64	6
4	32	2	8	8	32	8	64	8	1024	7
2	16	2	256	8	256	8	256	4	32	8
4	32	2	256	32	32	8	16	4	32	9
1	16	1	16	8	64	8	64	4	32	10
1	16	1	8	8	128	8	128	8	64	11
2	16	1	1024	64	256	64	128	8	512	12
4	128	0.5	32	32	64	8	64	4	32	13
2	16	1	1024	8	512	8	512	8	32	14
2	16	1	16	8	256	8	256	8	32	15
4	64	2	1024	16	32	4	32	8	128	16
2	32	2	8	32	256	16	128	8	64	17
2	32	2	32	32	64	32	64	32	64	18
4	32	2	32	8	64	8	32	8	64	19
4	16	2	128	8	512	8	128	8	64	20
4	64	2	128	128	1024	128	1024	512	1024	21
4	32	2	32	32	64	4	128	64	1024	22
4	32	2	256	8	64	8	64	8	64	23
4	32	2	64	32	64	16	32	8	64	24
1	4	2	128	32	64	16	64	8	64	25

The checkerboard titration method was employed to check if it was possible to overcome aminoglycoside resistance in Gram-negative bacteria (Table 6). Fortunately, aminoglycosides such, as amikacin and gentamicin still retained the ability to exhibit synergistic activity when used in combination with β-lactams (Table 6). This indicated that Gram-negative organisms are still sensitive to this antibiotic combination, as they were for more than thirty years [31], [32]. This synergism of aminoglycosides with β-lactams or quinolones could be due to the enhanced intracellular uptake of aminoglycosides facilitated by cell wall synthesis inhibitors that increase bacterial permeability [11].

**Table 6:** Effect of combinations of aminoglycosides on antibacterial activity by Checkerboard titration.

Number of isolates (%)								
Amikacin + Ciprofloxacin	Amikacin + Amoxicillin	Amikacin + Cephradine	Amikacin + Cefotaxime	Gentamicin + Ciprofloxacin	Gentamicin + Amoxicillin	Gentamicin + Cephradine	Gentamicin + Cefotaxime	Fractional Inhibitory concentration (FIC)
15 (100%)	10 (66.7%)	12 (80%)	13 (86.7%)	13 (86.7%)	8 (53.3%)	11 (73.3%)	11 (73.3%)	≤0.5 (Synergism)
0 (0%)	3 (20%)	2 (13.3%)	1 (6.7%)	1 (6.7%)	4 (26.7%)	1 (6.7%)	3 (20%)	>0.5-1 (Additive)
0 (0%)	2 (13.3%)	1 (6.7%)	1 (6.7%)	1 (6.7%)	3 (20%)	3 (20%)	1 (6.7%)	>1<4 (Indifferent)
0 (0%)	0 (0%)	0 (0%)	0 (0%)	0 (0%)	0 (0%)	0 (0%)	0 (0%)	≥4 (Antagonism)

In the clinic, there have been several reports of increased adverse effects in aminoglycoside-β-lactam combination therapy over β-lactam monotherapy [33], [34], [35], [36]. Thus, the decision for clinical implementation of empirical combination antibiotic therapy has to be taken with caution and should only be reserved for patients with resistant Gram-negative infections, when the benefits of synergistic antibiotic activity potentially outweigh the risks of increased side effects. Successful implementation of infection control measures is a must to reduce the problem of bacterial resistance, especially in countries where patients can have access to antibiotics without a prescription.
